# Impact of 6 months of treatment with intragastric balloon on body fat and quality of life in obese individuals with metabolic syndrome

**DOI:** 10.1186/s12955-017-0790-x

**Published:** 2017-10-24

**Authors:** Erika Paniago Guedes, Eduardo Madeira, Thiago Thomaz Mafort, Miguel Madeira, Rodrigo Oliveira Moreira, Laura Maria Carvalho de Mendonça, Amélio Fernando de Godoy-Matos, Agnaldo José Lopes, Maria Lucia Fleiuss Farias

**Affiliations:** 1 Division of Metabology, State Institute of Diabetes and Endocrinology (IEDE), Avenida das Américas, 2901, sala 305, Barra da Tijuca, Rio de Janeiro, RJ CEP 22631 001 Brazil; 2 Division of Gastroenterology, State University of Rio de Janeiro, Rio de Janeiro, Brazil; 3 Division of Pneumology, State University of Rio de Janeiro, Rio de Janeiro, Brazil; 4 Division of Endocrinology, Federal University of Rio de Janeiro, Rio de Janeiro, Brazil; 5 Division of Rheumatology, Federal University of Rio de Janeiro, Rio de Janeiro, Brazil

**Keywords:** Obesity, Health-related quality of life, Body composition, Intragastric balloon

## Abstract

**Background:**

Obesity is a worldwide public health issue with a negative impact on quality of life. Different weight loss interventions have demonstrated improvements in quality of life. The aim of this study was to investigate the effect of 6 months of treatment with an intragastric balloon (IGB) on health-related quality of life (HRQOL) and its relation to changes in body fat in obese individuals with metabolic syndrome (MS).

**Methods:**

Fifty obese patients with MS aged 18–50 were selected for treatment with IGB for 6 months. Body fat was assessed with anthropometric measures and dual-energy X-ray absorptiometry (DXA) at baseline and after removal of the IGB. HRQOL was evaluated with the short form of the World Health Organization Quality of Life (WHOQOL-BREF) at baseline and soon after removal of the IGB.

**Results:**

Thirty-nine patients completed the study. After 6 months, there was a significant improvement in quality of life (*p* = 0.0009) and health (*p* < 0.0001) perceptions, and in the Physical (*p* = 0.001), Psychological (*p* = 0.031), and Environmental domains (*p* = 0.0071). Anthropometric measures and total fat determined by DXA were directly and significantly related to an improvement in general aspects of quality of life. The decrease in the percentage of total fat was the parameter that better correlated with improvements in quality of life perception after regression (*p* = 0.032).

**Conclusions:**

In obese individuals with MS, weight loss parameters were associated with short-term improvements in HRQOL after 6 months of treatment with IGB. However, only total fat was independently related to HRQOL perception.

**Trial registration:**

ClinicalTrials.gov NCT01598233.

## Background

Obesity is one of the most important public health issues, and its incidence is increasing worldwide [1]. There is a clear association between obesity and several chronic diseases such as dyslipidemia, type 2 diabetes, cardiovascular and cerebrovascular diseases, sleep apnea, degenerative articulation disease, and several neoplasias [2, 3]. Overweight and obesity are also related to higher mortality rates [2, 4]. According to evidence, weight loss in obese adults appears to reduce morbidity and mortality [5, 6].

Health is a relevant determinant of a person’s quality of life, and this perception can be affected by aspects linked to the individual’s culture, religion, environment, and education [7]. In this way, health-related quality of life (HRQOL) is becoming an important health outcome indicator [7]. Different studies have demonstrated the relationship of weight excess and worse HRQOL [8–13]. Obesity-related pathologic conditions can contribute to HRQOL impairment in obese persons [8, 11]. Different social and psychological aspects can also contribute to a worse quality of life in overweight patients, as a concern for criticism, feelings of inferiority, low self-esteem, anxiety, and depression [9, 14, 15].

Different weight loss strategies are not only associated with clinical and psychological benefits but also with a better perception of quality of life [10, 16–24]. Intragastric Balloon (IGB) is one of the currently available strategies for weight loss with significant results in a short period of time. The IGB is placed in the stomach using endoscopy and left for up to 6 months, when it has to be removed due to durability. Therefore, most of the information on efficacy and safety about this device is limited to a short period of time. Improvements in general HRQOL have also been reported after treatment with IGB, but the effects of weight loss on specific domains remain to be better evaluated [25–27]. Body mass index (BMI) is the parameter most commonly used to evaluate the association of weight loss and quality of life, showing a negative relationship between these factors [9, 13]. However, BMI does not directly measure the proportion of fat [9]. The purpose of this study was to determine the effect of weight loss achieved with a 6-month treatment with IGB on HRQOL (including specific domains) in obese individuals with metabolic syndrome (MS) and its relationship with changes in body composition, including fat content measured by dual-energy X-ray densitometry (DXA).

## Methods

This study using IGB included a consecutive sample of 50 patients willing to lose weight, who sought treatment for obesity and MS and fulfilled the eligibility criteria for participation **from July/2011 to April/2012.** The study was registered at ClinicalTrials.gov (identifier: NCT01598233). The number of patients included in this study was defined by the number of IGB we received from Silimed Silicone Instrumental Médico Cirúrgico Hospitalar Ltda. The protocol was approved by the Ethics Committee of the State Institute of Diabetes and Endocrinology of Rio de Janeiro, where the patients were recruited, and written informed consent was obtained from each participant.

The criteria for inclusion in the study were age between 18 to 50 years, obesity (BMI ≥30 kg/m^2^), and MS based on International Diabetes Federation (IDF) criteria [28]. The exclusion criteria comprised type 1 or 2 diabetes mellitus, pregnancy, previous gastric surgery, hiatal hernia ≥5 cm, clotting disorders, potentially bleeding gastrointestinal lesions, alcoholism or use of drugs, previous history of psychiatric disorder, current use of antidepressants or other psychiatric drug, and weight loss treatment within the previous 6 months [29].

After the initial evaluation (week 0), a silicone IGB (Silimed Silicone Instrumental Médico Cirúrgico Hospitalar Ltda, Rio de Janeiro, RJ, Brazil) was implanted by upper gastrointestinal endoscopy under deep sedation. Under endoscopic visualization, the IGB was placed in the stomach and filled with 650 mL of normal saline solution (0.9% NaCl) and 20 mL of methylene blue solution. The patients were subsequently followed for up to 6 months when the IGB was then removed.

The follow-up visits were performed at weeks 0 (baseline), 8, 16, and 24. During each visit, the following anthropometric data were registered: body weight (kg) and height (m) for calculation of the BMI as weight in kilograms divided by the square of height in meters (kg/m^2^), and waist circumference (WC, cm), determined at the midpoint between the lowest rib and the iliac crest. The body fat content (%) was evaluated at weeks 0 and **24** by DXA using a Prodigy-GE densitometer (GE Healthcare, Inc., Madison, WI, USA).

Health-related quality of life (HRQOL) can be defined as an individual’s subjective perception of different aspects of life that are influenced by health status and includes subjective evaluations of physical functioning, mental health, and social/role functioning [10]. In this study, quality of life was measured by the short form of the World Health Organization Quality of Life (WHOQOL), the WHOQOL-BREF, an internationally widely used questionnaire to evaluate quality of life, translated and validated for the Brazilian population [30, 31]. This questionnaire evaluates and individual’s overall quality of life (question 1), general health (question 2), Physical Domain (pain, fatigue, energy, sleep, and rest), Psychological Domain (self-esteem, memory, positive and negative feelings, perceptions of body image, and appearance), Social Domain (assessment of personal relationships), and Environmental Domain (safety, financial resources, leisure time, home environment, transportation, convenience of getting information, and medical service) [9]. The WHOQOL-BREF questionnaire was applied to the study group at baseline and soon after removal of the IGB. WHOQOL-BREF was used with permission from WHO, even though the permission was obtained after the completion of the trial.

The statistical analysis was performed with GraphPad InStat 3.00 for Windows 95 (GraphPad Software, San Diego, CA, USA). We analyzed the mean absolute change in weight, BMI, WC, total fat (%), lean mass (in grams), and all domains from the WHOQOL-BREF from baseline to week 24 using Student’s *t* test for parametric variables and Wilcoxon matched pairs test for nonparametric ones. Pearson’s correlation coefficient or Spearman’s correlation coefficient was used to determine the correlations between variations in weight and body composition parameters and WHOQOL-BREF scores. Multiple linear regression was used to identify independent variables related to improvements in specific WHOQOL-BREF scores. The level of statistical significance was set at 5% (*p* ≤ 0.05).

## Results

Of the 50 patients included in the protocol, 11 did not complete the study due to gastric intolerance in four, balloon rupture in five, uterus cancer in one, and loss of follow-up in another one. Prospective data were analyzed for 39 patients who completed the study. The mean age of the cohort was 34.6 ± 7.1 years and the mean BMI was 40.0 ± 6.3 kg/m^2^. After 6 months of treatment with IGB, the mean reduction in weight, BMI, WC, Fat Free Mass and total body fat analyzed with DXA was 11.7 ± 9.6 kg (*p* < 0.0001), 4.4 ± 3.5 kg/m^2^ (*p* < 0.0001), 9.3 ± 8.2 cm (*p* < 0.0001), 3.7 ± 4.8 kg and 7.53 ± 7.62 kg (*p* < 0.0001), respectively.

Patients who completed the study also displayed a significant improvement in almost all aspects of HRQOL measured by the WHOQOL-BREF. These results are presented in Table 1. The only exception was the Social Domain, which presented only a trend toward significance.

**Table 1:** Effects of 6 months of treatment with an intragastric balloon on the participants’ quality of life, measured with the WHOQOL-BREF (completers)

	Baseline (Week 0; *n* = 50)	Post-treatment (Week 24, *n* = 39)	*p*
Question 1	57.2 ± 18.5 (20.0 – 100.0)	70.0 ± 13.7 (40.0 – 100.0)	<0.01
Question 2	49.6 ± 19.0 (20 – 100)	67.8 ± 16.4 (40.0 – 100.0)	<0.01
Physical Domain	54.3 ± 17.9 (14.2 – 92.8)	67.0 ± 16.2 (25.0 – 100.0)	<0.01
Psychological Domain	55.9 ± 17.2 (12.5 – 91.6)	64.5 ± 19.9 (16.6 – 95.8)	0.03
Environmental Domain	51.0 ± 14.0 (15.6 – 78.1)	58.6 ± 15.5 (12.5 – 87.5)	<0.01
Social Domain	60.5 ± 18.1 (25.0 – 91.6)	67.9 ± 20.3 (0.0 – 100.0)	0.05

Question 1 = “How would you rate your quality of life?”; Question 2 = “How satisfied are you with your health?”. Data are represented as mean ± standard deviation, except for questions 1 and 2, which are represented as median (min – max)

Correlation analysis was used to investigate whether the amount of weight loss would correlate with the improvement in different aspects of quality of life (Table 2). The reduction in all markers of excess weight (BMI, WC, weight, and total fat [%]) was directly related to the improvement in general aspects of HRQOL (questions 1 and 2 of the WHOQOL-BREF). These results were not observed for the specific domain of the questionnaires. For the Physical Domain, only the reduction in WC was associated with the improvement. For the Environmental Domain, only the reduction in body fat measured by DXA correlated with the improvement in the scores. Among all domains of the WHOQOL-BREF, “Physicological” did not meet the requirements.

**Table 2:** Correlation analysis between improvements in variables indicative of excess body fat and improvements in the WHOQOL-BREF domains (Δ for all variables)

	Question 1	Question 2	Physical domain	Psychological domain	Environmental domain
Anthropometric					
Δ BMI (kg/m^2^)	*r* = − 0.43	*r* = − 0.32	*r* = − 0.22	*r* = − 0.01	*r* = − 0.08
	*p* = 0.0061	*p* = 0.046	*p* = 0.17	*p* = 0.94	*p* = 0.62
Δ Weight (kg)	*r* = − 0.43	*r* = −0.34	*r* = − 0.24	*r* = − 0.02	*r* = − 0.07
	*p* = 0.005	*p* = 0.033	*p* = 0.13	*p* = 0.85	*p* = 0.66
Δ Waist (cm)	*r* = −0.49	*r* = −0.35	*r* = − 0.34	*r* = − 0.08	*r* = − 0.15
	*p* = 0.002	*p* = 0.03	*p* = 0.036	*p* = 0.62	*p* = 0.37
DXA					
Δ Fat Free Mass (g)	*r* = −0.36	*r* = −0.02	*r* = −0.04	*r* = 0.16	*r* = 0.30
	*p* = 0.04	*p* = 0.91	*p* = 0.79	*p* = 0.38	*p* = 0.09
Δ Total fat (%)	*r* = −0.61	*r* = −0.45	*r* = − 0.23	*r* = − 0.29	*r* = − 0.47
	*p* = 0.0002	*p* = 0.009	*p* = 0.19	*p* = 0.10	*p* = 0.0067

Question 1 = “How would you rate your quality of life?”; Question 2 = “How satisfied are you with your health?”. *BMI* Body mass index, *DXA* Dual-energy X-ray absorptiometry

Multiple linear regression was used to identify which variables (anthropometric and DXA) independently correlated with the improvements in quality of life. “Question 1” and “Question 2” were used as the dependent variable and three different factors indicative of weight excess as independent variables (i.e., BMI, WC, fat free mass and total fat [%]). Both models were also adjusted for age and gender. After regression, only total fat (%) remained independently related to question 1 (*p* = 0.047). On the other hand, no variable (i.e. BMI, WC, and total fat [%]) reached statistical significance after regression for Question 2.

## Discussion

In the present study, 6 months of treatment with IGB was associated with improvements in general and specific domains of HRQOL analyzed with the WHOQOL-BREF questionnaire. Body fat reduction, evaluated using DXA, was the only indicator of weight loss that was independently and directly related to improvements in general aspects of HRQOL. Considering the specific domains, only total fat measured by DXA was evidenced for the Environmental Domain. Therefore, it seems that other factors, rather than the amount of weight loss, are associated with improvements in the specific domains of HRQOL observed in obesity treatment with IGB.

Different authors have related overweight with a poor quality of life, and most previous studies of patients submitted to any obesity treatment have demonstrated improvement in general HRQOL [9, 14, 15, 17, 18, 32-34]. Hence, in addition to weight loss and improvement in comorbidities, evaluation of quality of life has been considered an important measure of success in obesity treatment [32, 34]. After a median follow-up of 9.6 years, an intensive lifestyle intervention for overweight/obese patients with type 2 diabetes was associated with a significantly lower decline in quality of life when compared with diabetes support and education alone [17]. Wu et al. compared four different weight loss interventions in a sample of 119 patients; after 6 months, the extent but not the type of intervention for weight loss (low-calorie diet suggestions [LCDS], LCDS plus sibutramine, LCDS plus orlistat, or very low-calorie diet) was highly correlated with favorable changes in HRQOL [34]. Weight loss above 5% of baseline values was necessary to show significant improvements in HRQOL in this study [34]. Studies analyzing quality of life after weight loss with bariatric surgery have shown a positive impact of weight loss on HRQOL [22–24].

The use of IGB is a nonsurgical and nonpharmacological strategy for weight loss and may be considered a kind of behavior therapy [26]. However, few studies have reported the impact of treatment with IGB on HRQOL [25–27, 35]. When compared with a control group, significant differential improvement in quality of life, measured with the Impact of Weight On Quality Of Life-Lite (IWQOL-Lite) questionnaire, was documented in 32 patients treated with IGB by month 6 [35]. In another study, improvement in obesity-related illnesses and quality of life was reported after weight reduction in 119 patients submitted to treatment with IGB for a period of 169.9 ± 34.8 days [26]. In a prospective controlled trial of 33 obese patients treated with the IGB for 6 months, quality of life also improved [27]. Our study demonstrated that weight loss, particularly the reduction in body fat, was associated with improvement in general HRQOL. These results strongly suggest that, although several different mechanisms may be proposed to explain the improvement in specific aspects of HRQoL after rapid weight loss, the reduction in body fat seems to be one of the main determinant in this improvement.

Pimenta et al. described obesity as a multifactorial condition implicating medical, psychiatric, and social aspects [9]. HRQOL is considered a multifactorial concept that links physical, psychological, and social aspects related to a particular disease or treatment [36]. Analysis from the WHOQOL-BREF questionnaire provides information about different domains, including physical (pain, sleep, and the capacity to perform daily activities), psychological (depression, self-esteem, and body image), social (relationships and social support), and environmental (physical safety with regard to access to transportation, leisure activities, and the availability of medical and social care) parameters [9]. Other quality of life questionnaires, such as the 36-item Short-Form Health Survey (SF-36) and the IWQOL-Lite, also include biopsychosocial domains [23, 35]. Despite studies relating obesity to negative impact on quality of life, or weight loss with improvement in quality of life, few have evaluated the relationship of weight loss parameters and quality of life domains [34].Weight loss over 5% with different clinical interventions was accompanied by improved SF-36 scores in the dimensions of physical functioning, role-physical, bodily pain, general health, role-emotional, and physical component in a Chinese study [34]. Bariatric surgery has been associated with improvement in biopsychosocial parameters of HRQOL [24]. Castro et al. compared two different types of IGB (Bioenterics BIB® and Heliosphere®) in 33 obese patients [25]. Twenty-seven patients answered the gastrointestinal quality of life index (GIQLI) and baseline total scores were similar for both treatment groups, with no differences at week 24 after balloon insertion. The physical dimension of the GIQLI in the group allocated to the Heliosphere® bag demonstrated a significant improvement, but no improvement was observed in terms of digestive symptoms, treatment effect, or emotional dimensions [25]. Physical and mental aspects were linked to quality of life in a Chinese obese population treated with IGB [26]. Our study demonstrated a positive impact of weight loss on specific domains of WHOQOL-BREF, except for the Social Domain, which revealed only a trend toward improvement.

A Brazilian study has demonstrated a negative impact of increased BMI on the perception of environmental characteristics [9]. Pan et al. suggested that body weight loss was somehow associated with the satisfaction in one’s environment [10]. The Environmental Domain evaluates aspects related to safety, opportunity to take leisure time, financial resources, the convenience of getting information and medical service, home environment, and transportation [10]. Surprisingly, an association between the amount of weight loss and improvements in specific HRQOL domains was only identified for the percentage of fat loss reduction (by DXA) and Environmental Domain. Recently, we demonstrated that the decrease in body fat percentage was the parameter that better correlated with improvements in the psychopathological profile in the same sample of patients included in the present study. However, we found no association between the amount of weight loss and the improvement in symptoms of depression [37]. It is worthy noticing that patients with a previous history of psychiatric disorders or current use of psychiatric medications were not included in this study. The exclusion of these patients was based in different points. First, it is impossible to determine how IGB would impact absorption of psychiatric medications; second, a significant number of psychiatric medications (including antidepressants and antipsychotics) can interfere with body weight, inducing either weight gain or weight loss; finally, weight gain and/or loss are somatic symptoms of several psychiatric syndromes. Therefore, it is important to state that the results of this study are not applicable to patients with psychiatric syndromes. Further studies are necessary to clarify the impact of IGB in this specific population.

Our study has a few limitations. First of all, this was a small and very selective population of obese patients with MS and further studies are necessary to confirm whether our findings would also be applicable to different populations (e.g., obese individuals without MS). The number of patient was defined based on the number of IGB we had available. Also, no information (i.e. DXA and WHOQOL-Bref) was collected from the 9 patients who had the IG balloon removed before 6 months. Since the main objective of the study was to investigate the impact of weight loss in HRQoL after 6 months, we believe that these patients should not be included in the analysis, particularly because some of them were excluded from the study before 2 weeks. Second, as only 10 individuals were male, we could not determine the impact of gender on the relationship between body composition and HRQOL. Third, a control group was not included in this study. Fourth, physical activity might be an important confounding variable in the relationship between weight loss and quality of life domains. Although we did not include a specific instrument to measure physical activity, this information was obtained in the initial and final evaluations. None of the participants was practicing regular physical activity in the 6 months prior to study enrollment, and this situation was sustained until the final visit. Therefore, we may speculate that physical activity may not have been an important determinant of our findings. Fifth, the IG balloon used in this study had to be removed after 6 months. New devices are being developed with longer durability (up to one year). Finally, we do not have follow-up data on this population.

## Conclusions

In conclusion, a 6-month treatment with IGB was associated with significant weight loss and improvements in general and specific domains of the WHOQOL-BREF. Fat mass reduction observed with DXA was the parameter that better correlated with improvement in general aspects of quality of life. The amount of weight loss or fat mass did not correlate with improvement in different domains of HRQOL, suggesting that other factors, rather than weight loss *per si*, can positively influence quality of life.
